# Chemical Profiling and Mechanistic Insights into Stichopodidae Viscus Extract for Ulcerative Colitis via UPLC-IMS-Q-TOF-HDMS^E^ and Network Pharmacology

**DOI:** 10.3390/ph19030470

**Published:** 2026-03-12

**Authors:** Liying Wang, Yinuo Liu, Nali Chen, Shanshan Xiao, Shuang Yang, Zhihua Lv

**Affiliations:** 1 Key Laboratory of Marine Drugs, Ministry of Education of China, NMPA Key Laboratory for Quality Research and Evaluation of Traditional Marine Chinese Medicine, School of Medicine and Pharmacy, Ocean University of China, Qingdao 266003, China; liyingwang@stu.ouc.edu.cn (L.W.); yinuoliu@stu.ouc.edu.cn (Y.L.); chennali@stu.ouc.edu.cn (N.C.); xiaoshanshan133@stu.ouc.edu.cn (S.X.); 2 Laboratory for Marine Drugs and Bioproducts, Qingdao Marine Science and Technology Center, Qingdao 266237, China

**Keywords:** UPLC-IMS-Q-TOF-HDMS^E^, ulcerative colitis, Stichopodidae Viscus, network pharmacology, metabolomics, serum pharmacochemistry

## Abstract

**Background:** The visceral organs of sea cucumbers belonging to the family Stichopodidae, also known as Stichopodidae Viscus (SV), have been traditionally used for the management of gastrointestinal disorders. Experimental evidence has shown that the ethanol extract of SV (SVE) alleviates ulcerative colitis (UC) symptoms in a mouse model. However, the chemical constituents of SVE and the potential molecular targets mediating its effects in UC remain unclear. **Methods:** In this study, SVE was prepared from *Apostichopus japonicus* (Selenka). A reliable and sensitive strategy integrating advanced analytical and informatics tools was employed to profile the chemical components of SVE. Analyses were performed using ultra-performance liquid chromatography coupled with ion mobility spectrometry and quadrupole time-of-flight mass spectrometry operating in high-definition MS^E^ (UPLC-IMS-Q-TOF-HDMS^E^), with data processed using the UNIFI scientific information system. Constituent identification relied on retention time (RT), accurate mass (MS1), experimentally acquired HDMS^E^ (MS2) spectra, and collision cross-section (CCS). Metabolomics-based approaches were further applied to characterize the in vivo exposure profile of SVE components in mouse serum and colon tissue after oral administration. Subsequently, the putative bioactive constituents and their underlying mechanisms of action were investigated using network pharmacology and molecular docking. **Results:** Based on the integrated identification strategy, a total of 78 compounds, including saponins, phenolic acids, fatty acids, and amino acids, were annotated in SVE, among which 6 compounds were verified using authentic reference standards to ensure unambiguous identification. Subsequently, 35 features in serum and 24 in the colon were found to be significantly altered following a single oral dose of SVE in mice, and were defined as SVE-related differential constituents. After network pharmacology analyses, 129 shared targets were identified between potential targets of SVE-related components in serum and UC-related targets, including PIK3CA, EGFR, and AKT1. Functional enrichment analysis suggested that SVE might exert its effects in UC through modulation of key nodes within the PI3K-Akt and EGFR signaling pathways, as well as lipid- and atherosclerosis-related pathways. Molecular docking results further indicated moderate binding affinities of representative SVE-related differential components toward PIK3CA, AKT1, and EGFR. **Conclusions:** This study clarifies the chemical basis and potential UC-related mechanisms of SVE, providing a scientific rationale for the development of SV-derived therapeutic candidates for UC.

## 1. Introduction

Stichopodidae Viscus (SV) is a marine-derived traditional medicine, referring to the viscera of sea cucumbers from the family Stichopodidae, including *Apostichopus japonicus* (Selenka), *Stichopus chloronotus* Brandt, and *Stichopus variegatus* Semper. Recorded in the Chinese Materia Medica for the treatment of gastrointestinal ailments (including gastric and duodenal ulcers), SV is a traditional marine remedy used in coastal regions of China. However, owing to their lower commercial value relative to the body wall, the sea cucumber viscera are often discarded as by-products during sea cucumber processing, despite constituting a substantial proportion of the organism’s biomass [1]. In contrast, the economically valuable sea cucumber body wall has attracted substantial research interest, with previous chemical investigations of sea cucumbers predominantly focusing on this tissue, which is rich in bioactive polysaccharides and peptides with anti-inflammatory and immunomodulatory activities [1,2,3,4]. Available evidence indicates that the body wall and viscera share certain classes of bioactive constituents, such as triterpene glycosides, while also exhibiting distinct chemical profiles, with tissue-specific distributions of saponins, lipids, fatty acids, and polysaccharides reported in different sea cucumber species [5]. In our previous study, oral administration of the ethanol extract of Stichopodidae Viscus (SVE) was shown to alleviate ulcerative colitis (UC) symptoms in a mouse model [6]. Nevertheless, the bioactive constituents responsible for this effect and their underlying mechanisms remain unclear.

Ultra-performance liquid chromatography coupled with ion mobility spectrometry and quadrupole time-of-flight mass spectrometry operating in high-definition MS^E^ (UPLC-IMS-Q-TOF-HDMS^E^) is an integrated platform combining chromatographic separation, ion mobility analysis, and high-resolution mass spectrometric detection. In addition to retention time (RT), accurate mass (MS1), and fragmentation patterns (MS2), collision cross section (CCS) values can be simultaneously measured, which reflect both ion size and three-dimensional conformation. This capability allows efficient separation and structural characterization of compounds in complex matrices [7,8]. This powerful technique, characterized by high resolution, sensitivity and accuracy, is widely applied in Traditional Chinese Medicine (TCM) research, including the characterization of chemical constituents [9] and identification of habitat-related chemical markers [10]. Furthermore, mass spectrometry-based metabolomics is widely applied in serum pharmacochemistry of TCM to investigate the dynamic changes in endogenous and exogenous metabolites under specific physiological or pathological conditions. This enables not only the screening of differential metabolites associated with pharmacological effects [11], but also the identification of absorbed compounds and their metabolites, offering critical insights into biotransformation and mechanisms of action of TCM [12,13].

To investigate the chemical basis of SVE, we profiled and annotated constituents in the SVE as well as the differential components observed in the serum and colon of mice following a single oral administration of SVE using UPLC-IMS-Q-TOF-HDMS^E^, with data processed through the UNIFI scientific information system within an untargeted metabolomics framework. Network pharmacology [14,15] and molecular docking [16] were subsequently applied to explore potential targets and provide insights into the possible mechanisms underlying the effects of SVE in UC.

## 2. Results and Discussion

### 2.1. Chemical Profiling and Annotation of SVE Constituents

**Figure 1:**
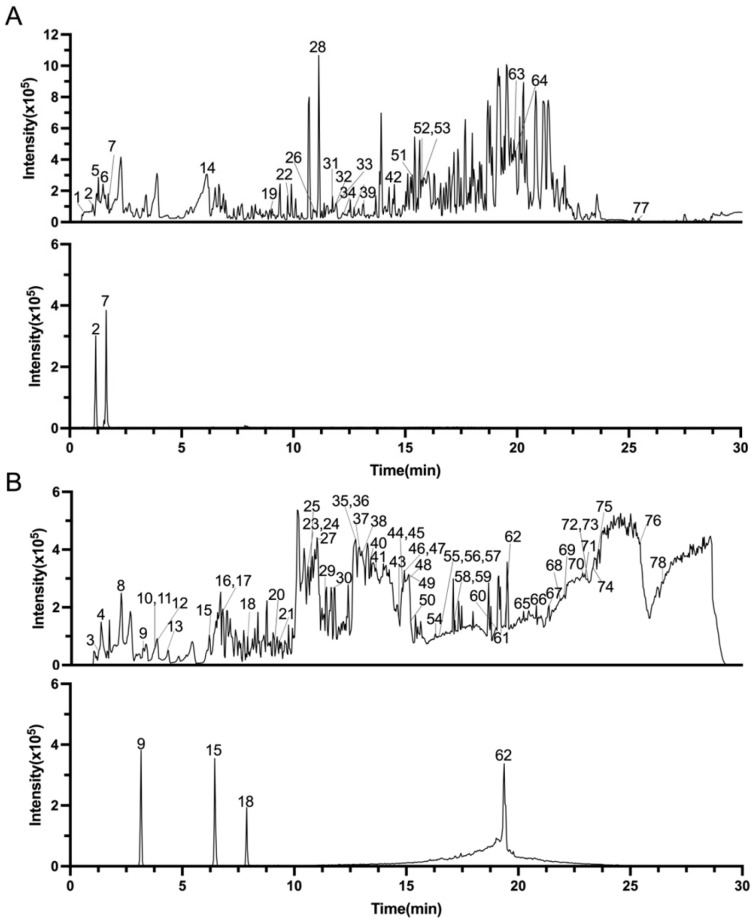
Base peak intensity (BPI) chromatograms of SVE. (**A**) BPI chromatogram in positive ion mode (above, SVE; below, standards) and (**B**) BPI chromatogram in negative mode (above, SVE; below, standards).

Base peak intensity (BPI) chromatograms of SVE obtained in positive and negative ionization modes are presented in Figure 1. The acquired MS data were processed using the UNIFI 3.6.0.21 scientific information system in combination with an in-house SV database (Table S1), enabling systematic MS-based annotation of SVE-related constituents.

Compound annotation was conducted via a comprehensive evaluation of MS1 data, experimentally acquired MS2 fragmentation patterns, RT, and CCS values, with additional manual verification and comparison to authentic reference standards where possible. A total of 78 SVE-related constituents were annotated, including 56 detected in negative ion mode and 22 detected in positive ion mode. All annotations were assigned confidence levels according to a four-tier identification system. Six constituents achieved Level 1 confidence through confirmation with reference standards; six components were assigned Level 2 confidence supported by literature-reported CCS values in combination with MS2 data; 43 constituents were classified as Level 3 based on diagnostic MS2 fragmentation patterns; and 23 compounds were assigned Level 4 confidence based on MS1 information only. Detailed mass spectrometric characteristics and annotation information are summarized in Table S2.

#### 2.1.1. Saponins

**Figure 2:**
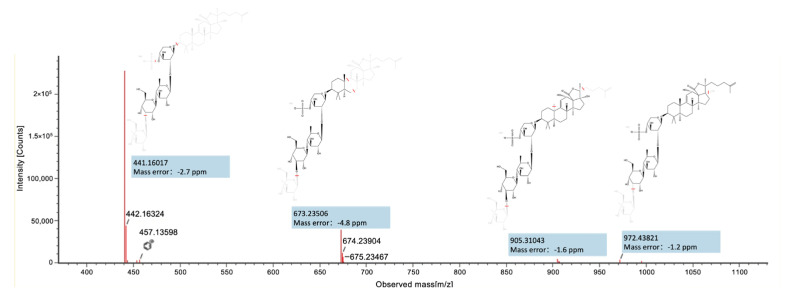
Typical MS2 fragmentation spectrum of compound (peak 35, annotated as scabraside A). The red lines mark the proposed bond cleavage sites.

A total of 33 triterpenoid saponins were annotated in SVE. These saponins exhibited characteristic MS2 fragmentation behaviors, featuring preferential cleavage of glycosidic bonds along with aglycone-related fragmentation pathways. Based on integrated evaluation of MS1 accurate mass, MS2 fragmentation patterns, and CCS values (Figure 2), peak 35 at 12.68 min was annotated as scabraside A (C_54_H_85_NaO_26_S, Level 3). In the negative ion mode, it displayed a molecular ion at *m*/*z* 1204.5012 and generated a fragment ion at *m*/*z* 972.4382 (C_47_H_72_O_19_S), corresponding to a neutral loss of 232.0630 Da (C_7_H_13_O_7_Na), which was attributed to a sodium atom, as well as the cleavage of a hydroxyl bond and the glycosidic bond accompanied by the loss of by an oxygen atom and an *O*-methyl-glucosyl residue (C_7_H_13_O_6_). Another characteristic fragment ion was observed at *m*/*z* 905.3104 (C_40_H_57_O_21_S), which was reasonably ascribed to the loss of a sodium atom, a CH_3_+H, a methyl-hexosyl (C_7_H_13_O_5_) moiety together with a hexenyl (C_6_H_11_) side-chain fragment. In addition, complete cleavage of the glycosidic linkage was proposed to produce a trisaccharide fragment ion at *m*/*z* 441.1602 (C_17_H_29_O_13_), consistent with a Glc-Rha-Xyl sequence (Glc, glucose; Rha, rhamnose; Xyl, xylose). During this fragmentation process, the precursor ion underwent an *O*-methyl-glucosyl residue (C_7_H_13_O_6_) and a SO_3_ loss, accompanied by cleavage of the triterpenoid aglycone moiety, ultimately yielding the corresponding trisaccharide fragment ion. These fragmentation pathways were proposed based on the experimentally acquired MS2 spectra. On the basis of the diagnostic fragmentation features, additional triterpenoid saponins were annotated.

#### 2.1.2. Fatty Acids

A total of 15 fatty acids were annotated in SVE. Saturated fatty acids typically exhibited characteristic MS2 fragmentation behaviors, primarily involving stepwise alkyl-chain cleavages accompanied by carboxyl-related neutral losses. Based on integrated evaluation of high-resolution MS2 fragmentation data, peak 68 (RT 21.98 min) was tentatively annotated as heptadecanoic acid (Level 3). In the negative ion mode, a predominant adduct ion [M+HCOO]^−^ at *m*/*z* 315.2537 was detected, which dissociated to generate the deprotonated molecular ion [M−H]^−^ at *m*/*z* 269.2488. A characteristic fragment ion at *m*/*z* 239.2381 was also observed, attributable to alkyl-chain-related fragmentation of the fatty acid. These MS2 features are consistent with those previously reported for saturated fatty acids, thereby supporting the proposed annotation.

Peak 69 (RT 22.19 min) was tentatively annotated as eicosapentaenoic acid (Level 3). In the negative ion mode, a deprotonated molecular ion [M−H]^−^ at *m*/*z* 301.2167 was observed, which underwent decarboxylation to generate a fragment ion at *m*/*z* 257.2275 corresponding to the neutral loss of CO_2_. On the basis of the diagnostic fragmentation features, the remaining fatty acids were annotated. They were assigned confidence Levels 2–4, depending on the availability of diagnostic MS2 fragmentation patterns and supporting literature information.

#### 2.1.3. Amino Acids

Nine amino acids were annotated in SVE. In the negative ion mode, D-phenylalanine (Level 1) produced a deprotonated molecular ion [M−H]^−^ that underwent characteristic fragmentation. The MS2 spectrum showed a prominent fragment ion at *m*/*z* 147.0447, which was assigned the molecular formula C_9_H_7_O_2_^−^. This fragment is attributable to cleavage of the C-N bond. By comparison with an authentic reference standard, D-phenylalanine was unambiguously identified in the SVE sample. Both RT (6.36 min vs. 6.45 min) and CCS values (135.77 Å^2^ vs. 136.51 Å^2^) were consistent within accepted tolerance limits, and the CCS value was different from that of L-phenylalanine (140.27 Å^2^). On the basis of these diagnostic MS2 fragmentation features, additional amino acids were annotated with confidence at Levels 1, 3, and 4.

#### 2.1.4. Others

In addition to the major compound classes described above, several minor constituents representing different chemical categories were detected in SVE, including esters, phenols, terpenoids, alkaloids and nucleobases.

As a representative example, 1-oleoyl-sn-glycero-3-phosphocholine (peak 62, Level 1) was identified based on comparison with an authentic reference standard. In the negative ion mode, a formate adduct ion [M+HCOO]^−^ at *m*/*z* 566.3459 was observed. A fragment ion at *m*/*z* 506.3254 (C_25_H_49_NO_7_P) was generated, which was attributed to the loss of a CH_3_ group from the phosphocholine moiety. The ion at *m*/*z* 435.2521 (C_21_H_40_O_7_P) is nitrogen-free and can be attributed to cleavage of the phosphocholine headgroup, involving dissociation of a choline-related N-containing moiety (C_5_H_13_N). In addition, the fragment ion at *m*/*z* 281.2484 can be attributed to dissociation involving loss of the glycerophosphocholine (GPC)-related fragment (C_8_H_19_NO_5_P), yielding a C18:1 fatty acyl carboxylate (C_18_H_33_O_2_). Comparison of the experimental RT (19.39 min) and CCS (CCS = 245.37 Å^2^) values with those measured for an authentic reference standard (RT 19.40 min, CCS 242.69 Å^2^) provided additional support for the annotation of 1-oleoyl-sn-glycero-3-phosphocholine.

Owing to the limited availability of reference standards, most of these compounds were tentatively annotated based on high-resolution MS2 fragmentation information.

### 2.2. Serum Pharmacochemistry of SVE

**Figure 3:**
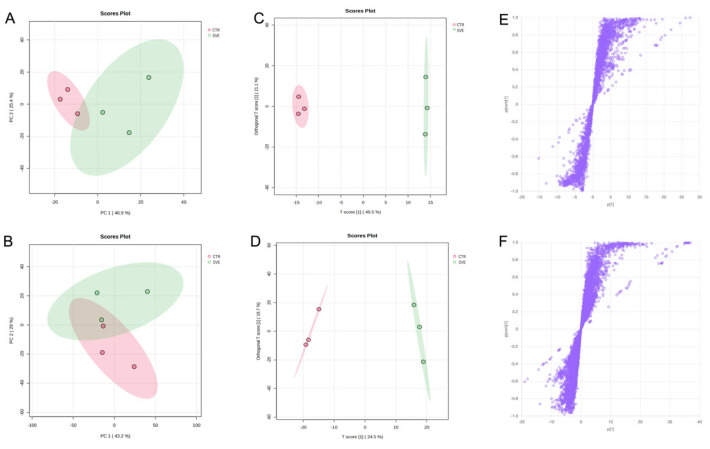
Multivariate statistical analysis of serum samples. PCA scatter plot in positive ion mode (**A**) and in negative ion mode (**B**). OPLS-DA plot in positive ion mode (**C**) and in negative ion mode (**D**). S-plot in positive ion mode (**E**) and in negative ion mode (**F**), where the purple circles represent individual variables.

Principal component analysis (PCA) was performed to explore global metabolic differences in the serum of mice treated with a single oral dose of SVE compared with the vehicle control (CTR) group (without SVE administration). Two-dimensional PCA score plots generated in positive and negative ionization modes are shown in Figure 3A,B. In both ionization modes, serum samples from the two groups were separated into distinct clusters, indicating pronounced differences in serum metabolic profiles following SVE administration. Metabolites contributing to this separation were therefore considered candidate serum-distributed constituents associated with SVE treatment.

Orthogonal projections to latent structures discriminant analysis (OPLS-DA) was further applied to characterize group discrimination. Consistent with the PCA results, the OPLS-DA models clearly distinguished the serum metabolic profiles of the SVE and CTR groups in both ionization modes (Figure 3C,D). Model validation based on 100 permutation tests showed satisfactory performance, with R^2^Y and Q^2^ values of 0.981 and 0.79 in positive ion mode, and 0.998 and 0.952 in negative ion mode, respectively, indicating good model robustness and predictive ability. Differential features contributing to group separation were visualized using S-plots (Figure 3E,F). Variables with greater distances from the origin were considered to contribute more significantly to group discrimination. Variable importance in projection (VIP) value was used to assess the contribution of individual features. Metabolites with VIP > 1.0 and *p* < 0.05 were selected as differential serum constituents. Based on these criteria, coupled with matching against public online databases and the in-house SV database, a total of 35 differential serum constituents were screened and annotated (Table S3).

### 2.3. Metabolomics-Based Analysis of Colon Constituents

**Figure 4:**
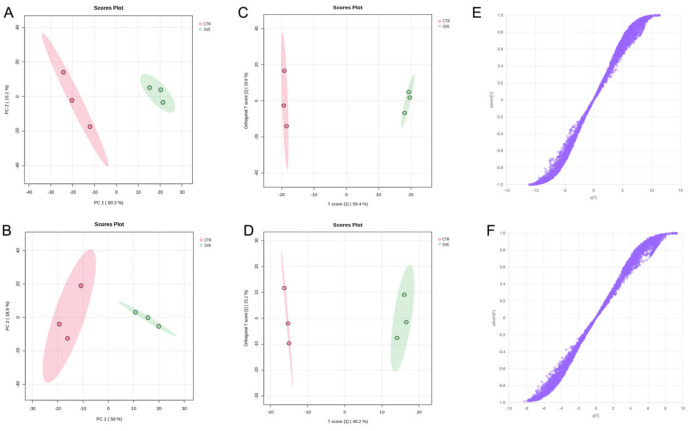
Multivariate statistical analysis of colon samples. PCA scatter plot in positive ion mode (**A**) and in negative ion mode (**B**). OPLS-DA plot in positive ion mode (**C**) and in negative ion mode (**D**). S-plot in positive ion mode (**E**) and in negative ion mode (**F**), where the purple circles represent individual variables.

PCA was performed to assess global metabolic differences in colon samples between the CTR and SVE groups. As shown in Figure 4A,B, data of colon samples from the two groups were clearly separated into distinct clusters in both ionization modes, indicating marked differences in colonic metabolic profiles following SVE administration. Metabolites contributing to this separation were therefore considered candidate SVE-related constituents distributed in the colon.

Consistent with the PCA results, OPLS-DA further distinguished the metabolic profiles in colon of the SVE and CTR groups in both ionization modes (Figure 4C,D). Based on 100 permutation tests, model validation was performed and yielded satisfactory performance. Specifically, the R^2^Y and Q^2^ values were 0.999 and 0.997 in positive ion mode, and 0.969 and 0.903 in negative ion mode, respectively, which confirmed the model’s robust reliability and excellent predictive capacity. Differential features contributing to group separation were visualized using S-plots (Figure 4E,F). Metabolite features with VIP values > 1.0 and *p* < 0.05 were selected as differential colon constituents. Following these criteria, and with support from database matching (including both public online databases and the in-house SV database), a total of 24 differential colon constituents were screened and annotated (Table S4).

### 2.4. In Vivo Tissue Distribution Profiles of SVE-Derived Constituents

Eight constituents in the SVE extract were traceable in the serum. They are amino acids (L-arginine and L-isoleucine), fatty acids (oleic acid), phenols (isoferulic acid), terpenoids (crassocolide I and dimethylcrocetin), glycerophospholipids (1-oleoyl-sn-glycero-3-phosphocholine), and carnitine-related compounds (deoxycarnitine). These extract-derived serum constituents likely represent bioavailable components absorbed into the systemic circulation following gastrointestinal exposure. In addition, serum-specific constituents may reflect metabolites formed during absorption and systemic metabolism.

Three SVE-related constituents detected in colon tissue, including L-methionine, uridine, and heronamide A, were also identified in the SVE extract, suggesting that these components can reach the colon in their prototype forms after oral administration. Together with other colon-specific differential features, these three extract-derived constituents indicate that some SVE-related components are preferentially retained or biotransformed in the intestinal environment and may be involved in local intestinal processes associated with UC.

Under the established analytical conditions, no common SVE-related constituents were detected in both serum and colon samples, suggesting distinct in vivo distribution profiles of SVE components. We inferred that some components in SVE may undergo structural modification during gastrointestinal absorption, hepatic metabolism, or intestinal microbial fermentation following oral administration.

Based on these tissue distribution characteristics, SVE-related constituents detected in serum were selected for subsequent network pharmacology analysis. This selection is justified by the fact that these components better reflect systemic exposure, thus providing a robust basis for investigating the potential systemic mechanisms associated with UC.

### 2.5. Network Pharmacological Target Prediction

**Figure 5:**
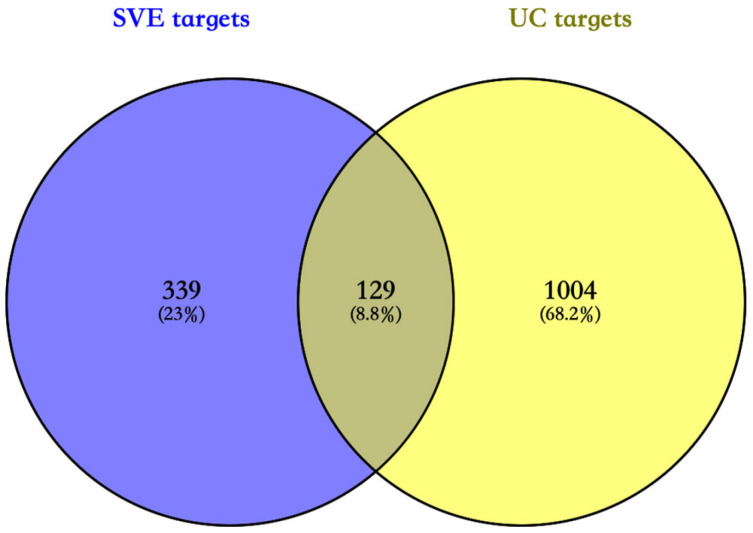
Venn diagram of predicted targets of serum-detected SVE-related constituents (SVE targets) and UC-associated targets (UC targets).

A total of 468 putative targets associated with serum-detected SVE-related constituents were predicted using SwissTargetPrediction, while 1133 UC-related targets were collected from public databases. Intersection analysis identified 129 overlapping targets between SVE-related and UC-associated targets (Figure 5).

**Figure 6:**
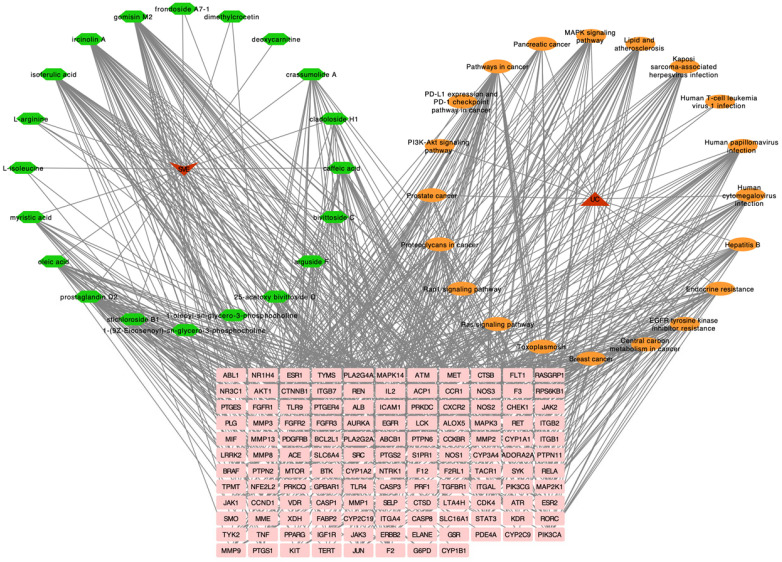
Constituent–target–pathway network. Green, orange, and pink nodes represent compounds, signaling pathways, and targets, respectively, while the red nodes represent the drug and disease. Edges represent the interactions between connected nodes.

The overlapping targets and corresponding SVE-related constituents were imported into Cytoscape 3.10.3 to construct a constituent–target disease–pathway network (Figure 6). Network topological analysis demonstrated that individual constituents targeted multiple proteins, and multiple constituents converged on common targets, reflecting the multi-constituent and multi-target regulatory characteristic of SVE.

**Figure 7:**
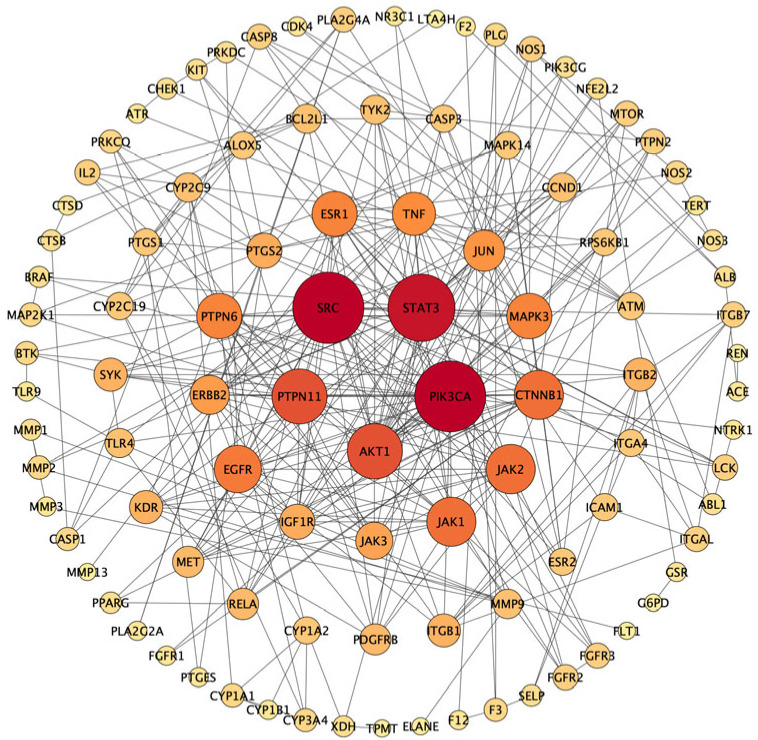
PPI network of the 129 overlapping targets. Nodes represent targets, with color intensity and size proportional to their degree. Edges represent interactions between two connected nodes.

To further explore target interactions, the 129 overlapping targets were imported into the STRING database to generate a protein–protein interaction (PPI) network (Figure 7), comprising 100 nodes and 324 edges. Based on degree ranking, SRC, AKT1, PTPN11, STAT3, and PIK3CA were identified as the top five hub targets. Notably, EGFR also exhibited a high degree value and was closely connected with multiple key targets, indicating its important role in the PPI network. Using median values of topological parameters as screening thresholds, 31 key targets were further identified (Table S5), which may be involved in UC-related biological processes regulated by SVE.

### 2.6. GO Enrichment and KEGG Pathway Analysis of the Core Targets

**Figure 8:**
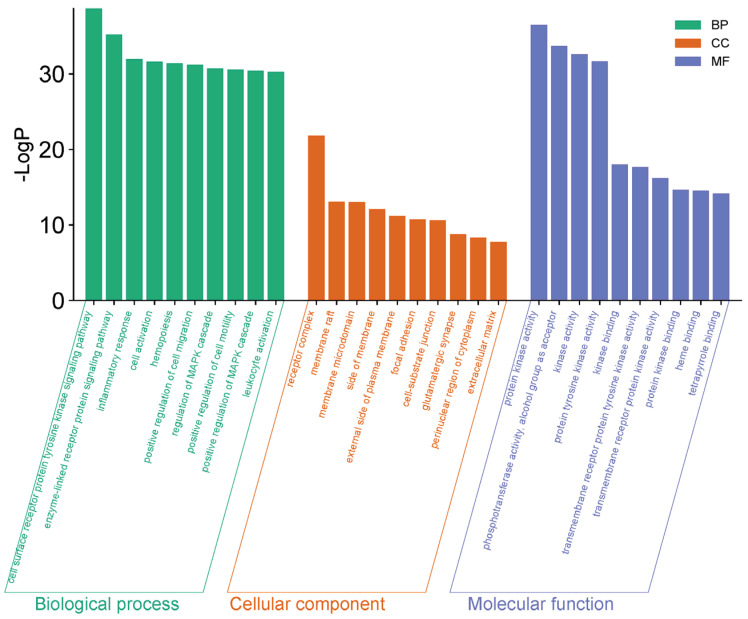
GO functional enrichment analysis of intersection targets.

Gene Ontology (GO) enrichment analysis of the core targets revealed significant enrichment across the Biological Process (BP), Molecular Function (MF), and Cellular Component (CC) categories, with the top ten terms in each category summarized in Figure 8. The enriched BP terms were mainly associated with receptor-mediated signaling, inflammatory responses, and cell activation. MF terms were predominantly related to protein kinase activity and phosphotransferase activity, whereas CC terms were enriched in receptor complexes, membrane rafts, and membrane microdomains.

**Figure 9:**
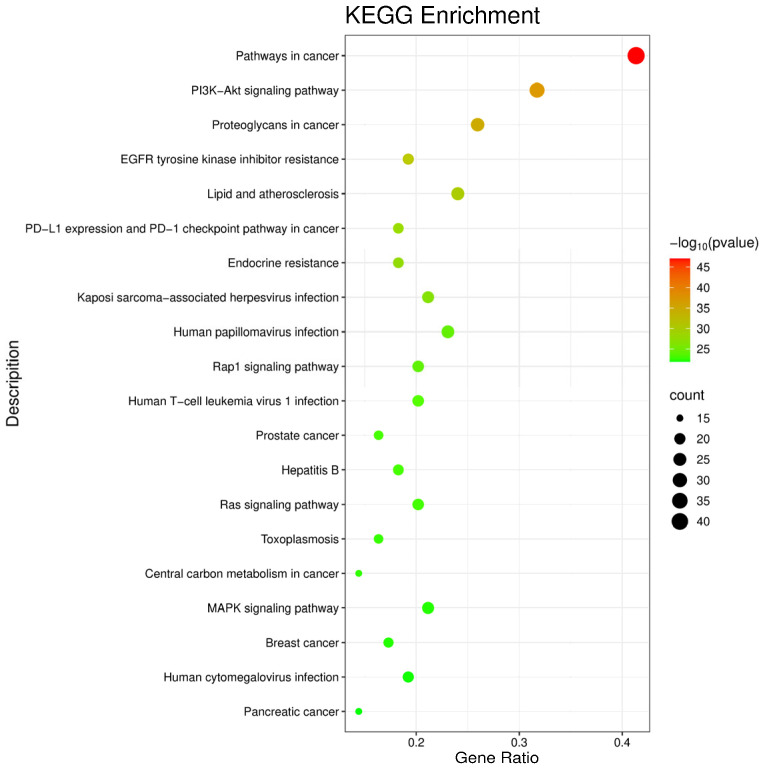
KEGG pathway enrichment analysis of intersection targets. Count refers to the number of genes associated with a specific pathway, with higher values indicating more genes involved. Gene Ratio represents the proportion of annotated genes in a given pathway relative to the total number of target genes. Color intensity is positively correlated with the statistical significance of pathway enrichment.

KEGG pathway enrichment analysis identified 20 significantly enriched pathways (*p* < 0.05), with the top-ranked pathways presented in Figure 9. Among these, pathways in cancer, the PI3K-Akt signaling pathway, proteoglycans in cancer, and EGFR tyrosine kinase inhibitor resistance were prominently enriched. Notably, the “pathways in cancer” category encompasses multiple fundamental cellular processes, including proliferation, apoptosis, and oxidative stress regulation, which are also implicated in UC pathogenesis [17]. In addition, the PI3K-Akt and EGFR signaling pathways have been widely reported to play important roles in intestinal inflammation and epithelial repair through regulation of immune and barrier functions [18,19].

Taken together, GO and KEGG enrichment analyses consistently identified the PI3K-Akt and EGFR signaling pathways as key regulatory axes mediating the effect of SVE on UC-related targets. The concordance between pathway enrichment results and the central positions of corresponding hub targets within the PPI network supports prioritization of these pathways for further experimental validation to elucidate the mechanistic basis of SVE-associated biological effects.

### 2.7. Molecular Docking Analyses

Based on network degree values, thirteen representative SVE-related constituents (25-acetoxy bivittoside D, arguside F, bivittoside C, caffeic acid, cladoloside H_1_, crassumolide A, gomisin M_2_, ircinolin A, isoferulic acid, myristic acid, oleic acid, prostaglandin D_2_, and stichloroside B_1_) were selected for molecular docking analysis. High-degree targets identified from the PPI network, including AKT1, MAP2K1, PIK3CA, MAPK3, and EGFR, were prioritized to evaluate potential interactions between candidate constituents and key UC-associated targets.

**Figure 10:**
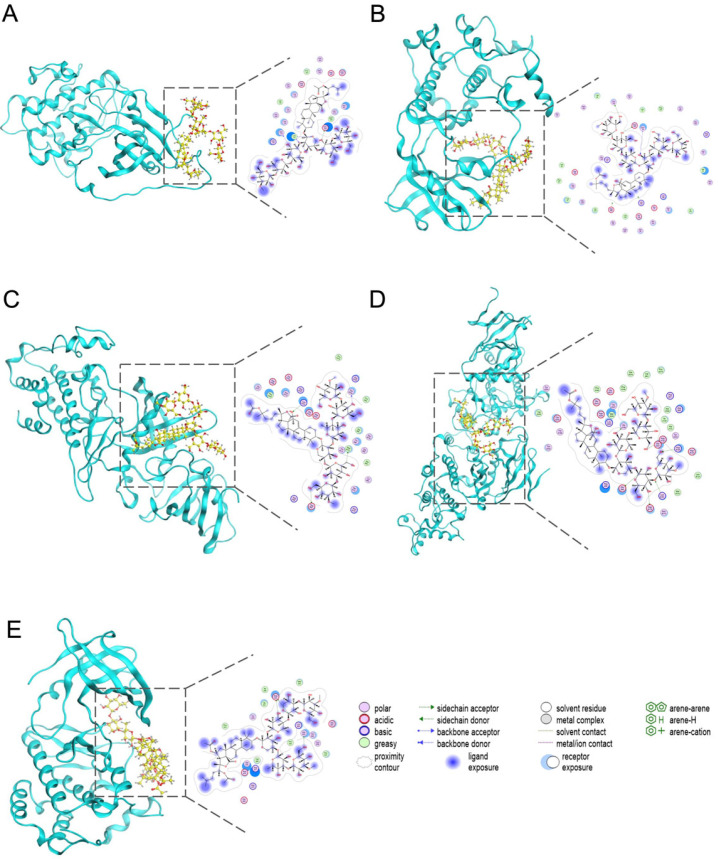
Molecular docking analysis of representative serum-detected SVE-related constituents with core targets. (**A**) Predicted binding pose of stichloroside B_1_ in AKT1; (**B**) predicted binding pose of 25-acetoxy bivittoside D in MAP2K1; (**C**) predicted binding pose of 25-acetoxy bivittoside D in PIK3CA; (**D**) predicted binding pose of 25-acetoxy bivittoside D in MAPK3; (**E**) predicted binding pose of cladoloside H_1_ in EGFR.

Docking results showed that most compound–target complexes exhibited binding energies below −5 kcal/mol [20], indicating moderate binding affinities. Docking poses were grouped according to receptor type, and the highest-scoring conformation for each target was selected for structural visualization and interaction analysis (Figure 10 and Table 1). However, these results were considered preliminary structural assumptions and required further experimental validation to confirm the binding affinities and biological relevance of the compound–target interactions.

## 3. Materials and Methods

### 3.1. Reagents and Chemicals

SV originating from *Apostichopus japonicus* (Selenka) was collected from Qingdao, Shandong, China, and authenticated by Professor Chuanyuan Tian (College of Fisheries, Ocean University of China, Qingdao, China). Its voucher specimen was deposited at the authors’ laboratory. Methanol, acetonitrile and water (LC-MS grade) were obtained from Merck Co., Ltd. (Darmstadt, Germany). Formic acid (LC-MS grade) and 1-oleoyl-sn-glycero-3-phosphocholine were purchased from Aladdin Reagents Co., Ltd. (Shanghai, China). The reference standards, including D-phenylalanine, L-isoleucine, D-valine, L-arginine and L-tryptophan, were obtained from Shanghai Yuanye Co., Ltd. (Shanghai, China).

### 3.2. Standards and Sample Preparation

Dried SV (40 g) was macerated in 400 mL of 70% (*v*/*v*) ethanol and subsequently extracted twice at 75 °C for 30 min per extraction. After filtration, the extract was concentrated to an extract-like paste using a rotary evaporator, then placed in an oven at 45 °C and dried until constant weight. SVE was weighed and dissolved in methanol to prepare a solution with a concentration of 50 mg/mL, which was filtered through a 0.22 μm filter membrane and used for subsequent analysis.

Six reference standards were dissolved in methanol. Before qualitative analysis, they were mixed to make a reasonable concentration and filtered through a 0.22 μm filter membrane.

### 3.3. Animal Experiments

All animal care and experimental procedures were performed in accordance with the National Guidelines for the Care and Use of Laboratory Animals and were approved by the Animal Ethics Committee of Ocean University of China (Approval No.: OUC-SMP-2025-04-03). Male C57BL/6 mice (SPF grade, body weight 20 ± 2 g) were purchased from Jinan Xingkang Biotechnology Co., Ltd. (Jinan, China; production license No. SCXK (Lu) 2022-0006). All mice were housed under a 12 h light/dark cycle in an environment with the relative humidity maintained at 50 ± 10% and the temperature kept at 22 ± 2 °C. They were allowed free access to water and chow diet. The mice were randomly divided into the CTR group and SVE group (*n* = 12 per group). The SVE dose corresponded to 1600 mg/kg (raw material equivalent). Three mice in the SVE group were euthanized at 1, 2, 3, and 4 h post-gavage (*n* = 3 per time point), and blood and colon samples were collected. The CTR group received the vehicle and was processed in parallel at the same time points.

### 3.4. Bio-Samples Preparation

#### 3.4.1. Serum Sample Preparation

Blood samples were kept at room temperature to allow clotting, followed by centrifugation at 4000× *g* for 10 min at 4 °C. The supernatant (serum) was collected for subsequent processing. Within each group, serum samples collected at 1, 2, 3, and 4 h were pooled, and three independent pooled samples were generated per group, each derived from four time points. These pooled samples (*n* = 3 per group) were used as biological replicates for subsequent metabolomics and statistical analyses. Ice-cold methanol (4 volumes, *v*/*v*) was added to precipitate proteins, and the mixture was vortexed for 30 s and incubated at −20 °C for 1 h. After centrifugation at 16,000× *g* for 15 min at 4 °C, the supernatant was collected and evaporated to dryness using a vacuum concentrator. The residue was reconstituted in 50% methanol (*v*/*v*) and centrifuged again at 16,000× *g* for 15 min at 4 °C. The final supernatant was transferred to injection vials for analysis.

#### 3.4.2. Colon Sample Preparation

Within each group, colon samples collected at 1, 2, 3, and 4 h were processed individually. To 30 mg tissue, cold methanol/water (4:1, *v*/*v*) at a volume of 450 μL, together with ceramic beads, was added. The samples were homogenized using a tissue homogenizer at 6000 rpm for 20 s per cycle, with three cycles in total. The homogenates were incubated at −20 °C for 1 h, followed by centrifugation at 16,000× *g* for 15 min at 4 °C. Equal volumes (200 μL) of the resulting supernatants from each time point were then pooled to generate three independent pooled samples per group, each derived from four time points. These pooled samples (*n* = 3 per group) were used as biological replicates for subsequent metabolomics and statistical analyses. The pooled supernatants were evaporated to dryness at 10 °C using a vacuum concentrator. The dried residue was reconstituted in methanol/water (1:1, *v*/*v*) and centrifuged at 16,000× *g* for 15 min at 4 °C. The final supernatant was transferred to injection vials, and a 4 μL aliquot was injected into the LC-MS system for analysis.

### 3.5. Chromatography and Mass Spectrometry Conditions

Chromatographic analysis was performed using a Waters ACQUITY™ UPLC system (Waters Corporation, Milford, MA, USA). Separation was achieved on a CORTECS UPLC T3 column (2.1 × 150 mm, 2.7 μm) maintained at 40 °C. The mobile phases consisted of water containing 0.1% formic acid (A) and acetonitrile (B). The flow rate was set at 0.3 mL/min. The gradient elution program was as follows: 0–4 min, 1% B; 4–23 min, 1–99% B; 23–26 min, 99% B; 26–27 min, 99–1% B; and 27–30 min, 1% B.

Mass spectrometric detection was carried out on a Waters SYNAPT XS mass spectrometer (Waters Corporation, Milford, MA, USA) equipped with an electrospray ionization (ESI) source operating in HDMS^E^ mode. Data were acquired in both positive and negative ion modes over an *m*/*z* range of 50–2000. The capillary voltage was set to 0.5 kV for positive ion mode and 2.0 kV for negative ion mode. The sampling cone voltage was 40 V. The source temperature and desolvation temperature were set to 120 °C and 500 °C, respectively, with a desolvation gas (nitrogen) flow rate of 1000 L/h and a cone gas flow rate of 50 L/h. Argon was used as the collision gas, and the collision energy was ramped from 20 to 45 eV. Mass calibration and real-time mass correction were performed using a LockSpray™ system with leucine enkephalin (100 pg/μL) infused at a flow rate of 10 μL/min, generating reference ions of *m*/*z* 556.2771 ([M+H]^+^) in positive ion mode and *m*/*z* 554.2615 ([M−H]^−^) in negative ion mode to ensure mass accuracy during data acquisition.

### 3.6. UNIFI-Based Data Processing and Compound Annotation

Information on the reported chemical constituents of SV was compiled through a comprehensive survey of publicly available databases, including CNKI, MEDLINE, PubMed, Web of Science, and ChemSpider. Based on this information, an in-house SV database was constructed using the UNIFI 3.6.0.21 scientific information system, incorporating compound names, molecular formulas, chemical structures, and exact masses [21]. In total, 573 candidate compounds were included, and detailed information is provided in Table S1. Raw HDMS^E^ data were processed and annotated using both UNIFI 3.6.0.21 and Progenesis QI with the in-house SV database and multiple supported databases, including MassBank, Atomax, Baoji Herbest Bio-Tech, DTP/NCI, Extrasynthese, Indofine, and MolBank. Compound matching was performed using predefined criteria, including an RT tolerance of ±0.1 min, a CCS tolerance of ±2.0%, and a mass error threshold of ±10 ppm. For positive ion mode, [M]^+^, [M+H]^+^, and [M+Na]^+^ ions were considered, whereas [M−H]^−^, [M]^−^ and [M+HCOO]^−^ ions were selected for negative ion mode.

Based on the integrated databases, mass spectrometric data acquired in HDMS^E^ mode were processed and annotated within UNIFI 3.6.0.21. Compound annotation was achieved through an integrated evaluation of RT, MS1, MS2, and CCS values, with confirmation by authentic reference standards when available.

### 3.7. Metabolomics-Based Profiling of SVE-Related Constituents in Mouse Serum and Colon

The acquired raw data were processed for peak alignment, deconvolution, and normalization, followed by statistical analysis as previously described [22]. Multivariate statistical analysis was then performed to compare the data of the CTR and SVE groups https://www.metaboanalyst.ca (accessed on 10 September 2025). PCA was initially applied to provide an overview of the metabolic profiles and assess global clustering patterns among samples. Subsequently, OPLS-DA was employed to enhance group discrimination between the two groups. Based on the OPLS-DA model, VIP values and S-plots were generated to identify differential features contributing to group separation. Features with VIP values > 1.0 and *p*-values < 0.05 were considered candidate differential components. Model validity was further evaluated using permutation testing, with R^2^Y and Q^2^ values used to assess model robustness. The screened differential components were subsequently subjected to structural annotation through database searches against public resources (including MassBank, Atomax, Baoji Herbest Bio-Tech, DTP/NCI, Extrasynthese, Indofine, and MolBank), in combination with the in-house SV database.

### 3.8. Network Pharmacology and Molecular Docking

#### 3.8.1. Prediction of Potential Targets and Construction of Component–Target Network

To predict potential targets associated with SVE-related components detected in serum, the SMILES strings of the selected compounds were retrieved from the PubChem database https://pubchem.ncbi.nlm.nih.gov (accessed on 10 September 2025) [23] and submitted to SwissTargetPrediction http://www.swisstargetprediction.ch (accessed on 10 September 2025) [24]. Predicted targets with a probability value of zero were excluded. The resulting component–target associations were used for subsequent network analysis.

#### 3.8.2. Screening of Relevant Genes for UC Targets in Drug Treatment

UC-related target genes were collected by querying the OMIM https://www.omim.org (accessed on 11 September 2025) [25], Therapeutic Target Database TTD; https://ttd.idrblab.cn (accessed on 11 September 2025) [26], and GeneCards https://www.genecards.org (accessed on 10 September 2025) databases [27] using “ulcerative colitis” as the keyword. Gene-level intersections between UC-related genes and the predicted component-associated targets were identified using the online tool Venny https://bioinfogp.cnb.csic.es/tools/venny/ (accessed on 11 September 2025). The overlapping genes were considered potential targets associated with the effects of SVE in UC.

#### 3.8.3. Protein–Protein Interaction Network Construction and Core Target Screening

The intersecting target genes were imported into the STRING database https://string-db.org (accessed on 11 September 2025) with the species restricted to Homo sapiens, a minimum interaction score set to >0.9, and isolated nodes removed [28]. The resulting protein–protein interaction (PPI) network was exported in TSV format and analyzed using Cytoscape 3.10.3. Network topological parameters, including degree centrality, betweenness centrality, closeness centrality, eigenvector centrality, network centrality, and local average connectivity, were calculated using the NetworkAnalyzer tool and the cytoNCA plugin, both of which are part of Cytoscape version 3.10.3. Nodes with higher centrality values were considered more important, and core target genes were subsequently identified.

#### 3.8.4. Construction of the Component–Target Disease–Pathway Network

Data corresponding to the intersecting target genes, serum-distributed components, and the top 20 significantly enriched pathways were imported into Cytoscape 3.10.3. Network topology was analyzed using the NetworkAnalyzer tool, and a regulatory interaction network illustrating the relationships among components, targets, UC, and pathways was constructed.

#### 3.8.5. GO and KEGG Enrichment Analysis

The intersecting SVE-related and UC-related target genes were subjected to Gene Ontology (GO) functional enrichment https://www.geneontology.org (accessed on 13 September 2025) and Kyoto Encyclopedia of Genes and Genomes (KEGG) pathway enrichment analyses https://www.genome.jp/kegg/ (accessed on 13 September 2025)using the Metascape platform https://metascape.org (accessed on 13 September 2025) [29]. For GO analysis, the top 10 enriched biological terms with the lowest *p*-values were selected and visualized as bar charts. For KEGG analysis, the top 20 significantly enriched pathways were selected and presented as bubble plots.

### 3.9. Molecular Docking

The three-dimensional crystal structures of the core target proteins were obtained from the Protein Data Bank (PDB; https://www.rcsb.org accessed on 15 September 2025). Protein structures were preprocessed by removing water molecules using MOE 2020.09 [30]. The structures of selected components were downloaded from the PubChem database https://pubchem.ncbi.nlm.nih.gov (accessed on 15 September 2025) in Mol2 format. Molecular docking was performed using MOE 2020.09 [31], and binding affinity was evaluated based on docking scores, with lower (more negative) scores indicating stronger binding between ligands and target proteins.

## 4. Conclusions

In conclusion, a total of 78 SVE-related constituents were annotated. Following a single oral administration of SVE in mice, 35 and 24 differential constituents were detected in serum and colon tissues, respectively. Network pharmacology analysis predicted that the constituents in serum may be associated with 129 UC-related targets and multiple pathways, including cancer-related pathways, the PI3K-Akt signaling pathway, lipid- and atherosclerosis-associated pathways, and the EGFR signaling pathway. Among these targets, PIK3CA, AKT1, and EGFR emerged as central nodes consistently highlighted by pathway enrichment and PPI network topology, suggesting that they represent priority targets for subsequent investigation.

Nevertheless, the functional roles of these predicted targets and signaling pathways warrant further experimental validation, particularly in the context of pathological UC conditions. Future pharmacological and molecular studies investigating PIK3CA-, AKT1- and EGFR-mediated signaling pathways will be critical to elucidate their regulatory roles, as well as the biological basis and potential therapeutic application of SVE for UC research.

## Figures and Tables

**Table 1:** Molecular docking results of binding energies between core SVE-related constituents and key targets.

SVE	Binding Energy (kcal/mol				
	AKT1	MAP2K1	PIK3CA	MAPK3	EGFR
25-acetoxy bivittoside D	−10.35	−11.69	−10.53	−11.35	−10.09
arguside F	−10.42	−9.75	−9.26	−9.50	−10.47
bivittoside C	−8.73	−9.59	−9.31	−10.66	−10.12
caffeic acid	−4.80	−5.06	−5.43	−4.79	−4.96
cladoloside H_1_	−9.49	−10.58	−9.16	−10.53	−11.06
crassumolide A	−5.87	−6.36	−5.82	−6.76	−5.79
gomisin M_2_	−6.94	−7.03	−6.14	−7.17	−6.15
ircinolin A	−7.28	−7.16	−7.39	−7.27	−7.06
isoferulic acid	−5.10	−5.31	−5.02	−5.18	−5.04
myristic acid	−5.94	−6.16	−6.53	−6.52	−6.92
oleic acid	−6.54	−6.36	−7.17	−6.99	−6.16
prostaglandin D_2_	−6.60	−7.49	−7.64	−6.99	−6.48
stichloroside B_1_	−11.14	−10.55	−9.80	−10.52	−10.91

## Data Availability

The original contributions presented in this study are included in the article/Supplementary Material. Further inquiries can be directed to the corresponding authors.

## Supplementary Materials

The following supporting information can be downloaded at https://www.mdpi.com/article/10.3390/ph19030470/s1. Table S1: In-House Database of SV-Related Constituents; Table S2: Annotated Constituents in SVE Based on UNIFI Analysis; Table S3: Annotated SVE-Related Constituents Detected in Serum Samples; Table S4: Annotated SVE-Related Constituents Detected in Colon Samples; Table S5: Important Genes in Protein–Protein Interaction Network.
